# Relationship between different PANSS cognitive factors and cognition assessed with MCCB in patients with first psychotic episode of schizophrenia

**DOI:** 10.1192/j.eurpsy.2024.609

**Published:** 2024-08-27

**Authors:** R. Rodriguez-Jimenez, Á. Sánchez-Cabezudo, M. Scala, L. García-Fernández, L. Sánchez-Pastor, D. Rentero, I. Martínez-Gras, M. Caballero, J. M. Espejo-Saavedra, A. Nuñez-Doyle, O. Jiménez-Rodríguez, A. I. Aparicio-León, J. L. Santos

**Affiliations:** ^1^Psychiatry, Instituto de Investigación Hospital 12 de Octubre (imas12); ^2^CIBERSAM, Madrid, Spain; ^3^Department of Biomedical and Neuromotor Sciences (DIBINEM), University of Bologna, Bologna, Italy; ^4^Psychiatry, Universidad Miguel Hernández, Alicante; ^5^Psychiatry, Hospital Virgen de la Luz, Cuenca, Spain

## Abstract

**Introduction:**

The Positive and Negative Syndrome Scale (PANSS) has been used as a universal instrument for clinical assessment of psychopathology in schizophrenia. Different studies have analyzed the factorial structure of this scale and have suggested a five-factor model: positive, negative, excited, depressive, and cognitive/disorganized factors. Two of the most used models are the Marder´s solution and the Wallwork´s one.

**Objectives:**

The aim of this work was to study the correlations of the two cognitive factors (Marder and Wallwork) with a cognitive assessment performed with a standard cognitive battery, in a sample of patients with first psychotic episode of schizophrenia.

**Methods:**

Seventy four patients with first psychotic episode of schizophrenia (26.9, SD:7.8 years old; 70.3% male) were included. The cognitive assessment was performed with the MATRICS Consensus Cognitive Battery (MCCB). The MCCB present seven cognitive domains: Speed of processing, Working memory, Attention/Vigilance, Verbal Learning, Visual Learning, Reasoning and Problem Solving, and Social cognition). Pearson correlations were performed between MCCB scores and Marder´s PANSS cognitive factor (P2, N5, G5, G10, G11, G13, G15) and Wallwork´s one (P2, N5, G11).

**Results:**

Correlation between MCCB scores and cognitive factors of Marder and Wallwork can be seen in the table.

**Table:** 

	Marder´s cognitive factor	Wallwork´s cognitive factor
Speed of processing	r = -0.461; p<0.001	r = -0.455; p<0.001
Attention/Vigilance	r = -0.414; p<0.001	r = -0.415; p<0.001
Working memory	r = -0.449; p<0.001	r = -0.468; p<0.001
Verbal Learning	r = -0.511; p<0.001	r = -0.405; p<0.001
Visual Learning	r = -0.252; p=0.024	r = -0.254; p=0.029
Reasoning and Problem Solving	r = -0.244; p=0.036	r = -0.272; p=0.019
Social cognition	r = -0.268; p=0.024	r = -0.202; p=0.091

**Conclusions:**

Both PANSS cognition factors show a moderate correlations with Speed of processing, Working memory, Attention/Vigilance and Verbal Learning assessed by MCCB. More discrete correlations were found with Visual Learning, Reasoning and Problem Solving, and with Social cognition (in fact, non-significant correlation with Wallwork´s cognitive factor was found).

Acknowledgements. This study has been funded by Instituto de Salud Carlos III (ISCIII) through the project PI19/00766 and co-funded by the European Union.

**Disclosure of Interest:**

None Declared

